# Assessment of Pulpal Status in Primary Teeth Following Direct Pulp Capping in an Experimental Canine Model

**DOI:** 10.3390/diagnostics12082022

**Published:** 2022-08-21

**Authors:** Andreea Igna, Cornel Igna, Mariana Ioana Miron, Larisa Schuszler, Roxana Dascălu, Mihaela Moldovan, Adrian Aristide Voicu, Carmen Darinca Todea, Marius Boariu, Maria-Alexandra Mârțu, Ștefan-Ioan Stratul

**Affiliations:** 1 Department of Pediatric Dentistry, Pediatric Dentistry Research Center, Faculty of Dental Medicine, “Victor Babes” University of Medicine and Pharmacy, 300041 Timisoara, Romania; 2 Department of Surgery, Faculty of Veterinary Medicine, Banat University of Agricultural Sciences and Veterinary Medicine “Regele Mihai I al Romaniei”, 300645 Timisoara, Romania; 3 Department of Oral Rehabilitation and Dental Emergencies, Interdisciplinary Research Center for Dental Medical Research, Lasers and Innovative Technologies, Faculty of Dental Medicine, “Victor Babes” University of Medicine and Pharmacy, 300041 Timisoara, Romania; 4 Department of Imagistic Diagnosis, Faculty of Veterinary Medicine, Banat University of Agricultural Sciences and Veterinary Medicine “Regele Mihai I al Romaniei”, 300645 Timisoara, Romania; 5 Department of Pathological Anatomy, Municipal Emergency Clinical Hospital, 300231 Timisoara, Romania; 6 Department of Functional Sciences, Faculty of Medicine, “Victor Babes” University of Medicine and Pharmacy, 300041 Timisoara, Romania; 7 Department of Endodontics, Faculty of Dental Medicine, TADERP Research Center, “Victor Babes” University of Medicine and Pharmacy, 300041 Timisoara, Romania; 8 Department of Periodontology, Grigore T. Popa University of Medicine and Pharmacy, 700115 Iasi, Romania; 9 Department of Periodontology, Faculty of Dental Medicine, Anton Sculean Research Center for Periodontal and Peri-Implant Diseases, “Victor Babes” University of Medicine and Pharmacy, 300041 Timisoara, Romania

**Keywords:** vitality pulp testing, pulse oximetry, LDF, pulp capping

## Abstract

(1) Background: This study aimed to assess the pulpal response of primary teeth by pulse-oximetry (PO) in a canine model, following direct pulp capping (DPC). (2) Methods: Forty-eight primary teeth from eight canine subjects were divided into three treatment groups, based on the DPC material—calcium hydroxide (CH), MTA, Biodentine^TM^)—and three corresponding control groups. Data from PO pulp testing were correlated with laser Doppler flowmetry (LDF) testing, computer tomographic (CT) densitometry and histological analysis; the experiment lasted 14 days. (3) Results: SpO₂ recordings revealed statistically significant differences (*p* = 0.002, <0.05) between the treatment and control groups, and no significant differences (*p* = 0.257, >0.05) were observed between treatment groups. LDF recordings showed significant differences (*p* = 0.002, <0.05) between the treatment and control groups and identified significant differences between materials (*p* = 0.001, <0.05). CT densitometry indicated vital pulps in all teeth, with pulpal inflammation detected in 6/8 CH-capped teeth and 2/8 MTA-capped teeth. Histologic evaluation confirmed vital pulp in all specimens, with different degrees of inflammation. (4) Conclusions: Within its limitations, the present study confirms the diagnostic value of PO evaluation of pulpal status in primary teeth with histologic means after pulp-capping procedures in a canine model. However, various degrees of pulpal inflammation elicited by different pulp-capping materials seem not to correlate with the obtained PO values.

## 1. Introduction

Dental trauma and deep carious lesions are leading causes of pulp disease in primary teeth. The primary goal of pulp therapy is to maintain the integrity and health of the teeth and their supporting tissues while maintaining the vitality of the pulp [1]. The prognosis of teeth that underwent vital pulp therapy (VPT) procedures is evaluated on the basis of clinical symptoms, percussion, radiographic examination, and pulp testing [2]. Diagnostic pulp tests are, therefore, an essential aid in determining the outcome of various pulp-capping procedures. Pulp testing provides information about the pulp status by investigating either the neural component (sensibility tests) or the vascular supply (vitality tests) [3]. Studies have shown that the pulp’s vitality depends on the health of its vascular supply [4]. The vital pulp may erroneously test nonvital (false negative response) relative to sensibility tests in cases when only its neural component is injured, such as in recently traumatized teeth. Inversely, pulp nerve fibers are more resistant to necrosis than the vascular tissue; thus, sensibility tests may produce false positive results in such a situation [5]. Performing sensibility tests in primary teeth is particularly challenging because it may compromise cooperation with the child, and the results of the tests are highly subjective depending on the patient’s ability to understand the situation [6]. Vitality tests have been introduced in practice to assess the parameters that characterize the vascular supply of the dental pulp, which is the true determinant of the pulp’s vitality [4]. Thus vitality pulp testing is considered an objective method that requires no subjective responses from the patient and offers a more reliable result compared to sensibility testing [7]. 

Among vitality pulp testing methods, pulse-oximetry (PO) and laser Doppler flowmetry (LDF) proved their ability to differentiate, with high accuracy, vital from nonvital teeth [8]. To our knowledge, there are no studies investigating pulse-oximetry for the monitorization of pulp status in primary teeth following pulp capping. Despite its precision, the routine use of LDF in clinical settings is hindered by the method as it is expensive, technique-sensitive and lengthy [9]. PO, on the other hand, is carried out using a simpler device—a conventional pulse oximeter, but with modified sensor holders [10,11,12]—due to the fact that a dedicated PO device/sensor for dental use is not yet available [13]. Studies on PO in primary teeth are limited, and most of them were performed in anterior teeth [12,14] due to the physical limitation of bulky sensors. Nevertheless, the numerous advantages of PO (non-invasiveness, relatively cheap cost, simple technique and painless procedure) [13] would make it ideal for routine use in children, for both the initial diagnosis and follow-up of vital pulp treatments. For veterinary use, only one study has so far investigated PO to detect blood flow in vital canine teeth [15]. 

Over the last decades, research in the field of biomaterials and pulp biology have brought concepts such as conservative pulp treatment and regenerative endodontics into attention. Indications of VPT have now expanded to mature teeth with signs and symptoms indicative of irreversible pulpitis [16]. Calcium hydroxide (CH), Mineral Trioxide Aggregate (MTA) and Biodentine^TM^ (Septodont Ltd., ST MAUR DES FOSSES, France) are among the most popular VPT biomaterials used today [17]. CH was regarded as the gold standard for VPT for a long period of time. Although numerous studies have revealed drawbacks such as changes in the physical structure of dentin, pulp surface inflammation [18] and the induction of sterile pulp necrosis areas at the contact between CH and the vital pulp tissue [19], it is still widely used in clinical practice. Calcium-silicate cements, such as MTA and Biodentine^TM^, induce improved pulp responses, promoting a rapid pulp healing with minimal inflammation [20]. These materials are currently used in primary teeth in various vital pulp therapy procedures (indirect pulp treatment, indirect/direct pulp capping and pulpotomy) [21,22].

The purpose of this study was to assess the pulpal response of primary teeth by pulse-oximetry in a canine model, following direct pulp capping with calcium hydroxide, MTA and Biodentine^TM^. The data from pulse oximetry pulp testing were correlated with LDF pulp testing, radiographical and computer tomography (CT) imaging and histological analyses. The first null hypothesis was that pulse-oximetry cannot provide a confirmation of the pulp’s status (vital/non-vital) in primary teeth following direct pulp capping. Regarding pulp-capping materials, a second null hypothesis was formulated, stating that there is no statistically significant difference between the pulpal response generated by calcium hydroxide and calcium silicate-based materials. 

## 2. Materials and Methods

The study was carried out at the Banat University of Agricultural Sciences and Veterinary Medicine “Regele Mihai I al Romaniei” from Timisoara (USAMVBT), in collaboration with the University of Medicine and Pharmacy “Victor Babes” Timisoara (UMFVBT). The experiment was approved by the ethics committees of both universities (approval nr. 133/07/2020 of USAMVBT, approval nr. 51/2020 of UMFVBT) and by the local Sanitary-Veterinary and Food Safety Authority (approval nr. 24876/10.08.2020). No animals were harmed during the procedures. 

The study lasted two weeks and included primary teeth from eight canine subjects (aged three months old, belonging to the same litter) and was concluded with the extraction of three primary teeth/subject, which were soon replaced by permanent successors. All in vivo interventions (direct pulp capping, vitality tests and imagistic research) were performed under general anesthesia. 

### 2.1. Operative Procedures

The selected teeth—mandibular canines (804), mandibular second premolars (806) and third premolars (807)—were divided into 6 groups, based on the capping material that will be used: the calcium hydroxide group (Dycal^®^, Dentsply Sirona, Charlotte, NC, USA)—the 804 s; the MTA group (Bio-MTA Plus, PPH CERKAMED Wojciech Pawłowski, Stalowa Wola, Poland)—the 806 s; the Biodentine^TM^ group (Biodentine^TM^, Septodont Ltd., France)—the 807 s; and the corresponding control group—704 s, 706 s and 707 s. The direct pulp-capping procedure was performed under general anesthesia. The anesthesia protocol consisted of premedication with xylazine (1 mg/kg b.w., i.v.) and ketamine (5 mg/kg b.w., i.v.) followed by induction with propofol (3 mg/kg b.w., i.v.); general anesthesia was maintained with isoflurane vaporized in oxygen using intermittent positive pressure ventilation; postoperative analgesia was provided with one dose of butorphanol (0.4 mg/kg b.w., s.c.) administered 15 min before recovery. No other medication was administered in the following period during the experiment. Rubber dam was used to isolate the teeth from the oral environment. Before cavity preparation, the teeth were disinfected using 2.5% sodium hypochlorite. The cavities were prepared on the buccal surface of each tooth. A standardized pulp exposure of 1 mm in diameter was performed with a 010 round diamond bur at high speeds under thorough water irrigation. Hemorrhaging was controlled by light pressure with moist saline cotton pellets before placement of the pulp-capping material. Pulp-capping materials were applied in each cavity using Dovgan carriers and round condensers, and the final restoration was completed with conventional glass ionomer cement (GC EQUIA Forte Fill-capsules, GC Europe). At 14 days post-intervention, the treated teeth were extracted under general anesthesia, as atraumatic as possible, by a designated veterinary surgeon (C.I.) in the Department of Small Animal Surgery of USAMVBT. 

### 2.2. Complementary Research

Pulp vitality was assessed prior to (T0) and after the direct pulp-capping procedure at 24 h (T1), 7 days (T2) and 14 days (T3) by PO and LDF. Inclusion criteria: intact healthy primary teeth (clinically and radiographically) and teeth with adequate size for sensor placement (upper and lower canines, second and third premolars); exclusion criteria: fractured teeth, teeth with calculus accumulation and small teeth not suitable for sensor placement (e.g., incisors and first premolars). 

**Figure 1:**
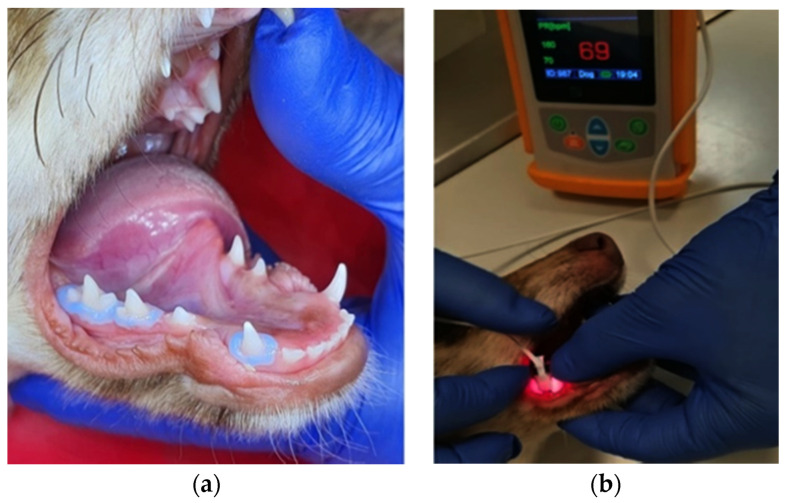
SpO_2_ recording in a canine subject: (**a**) liquid rubber dam isolation of teeth 804, 806 and 807 before SpO_2_ recording; (**b**) SpO_2_ recording in tooth 807 in low light conditions.

The pulse-oximetry measurements were carried out using a pulse-oximeter designed for veterinary use (SOMO PO100VET, SOMO International CO., Ltd., Hong Kong, China), with a compatible nasal alar sensor (Nasal Alar Fast SpO₂ Sensor, Koninklijke Philips N.V., Amsterdam, the Netherlands) of an appropriate size for dental use, for both anterior and posterior teeth. It uses red (640 nm) and infrared (940 nm) wave lengths to transilluminate the tissue and to detect absorbance peaks due to pulsatile blood circulation, and it calculates the pulse rate and oxygen saturation. The gingival margin was isolated using a liquid rubber dam prior to the placement of the sensor on the tooth (Figure 1). The sensor was hand-stabilized on the tooth with the emitter on the buccal side of the tooth and the receptor on the lingual side. The oxygen saturation (SpO₂) of the dental pulp was registered in low-light conditions. The initial measurements were performed in all healthy primary teeth that met the inclusion criteria: upper and lower canines (504, 604, 704 and 804), upper and lower second premolars (506, 606, 706 and 806) and third premolars (507, 607, 707 and 807). Following DPC, SpO₂ measurements were repeated for the treated teeth (804 s, 806 s and 807 s) and the corresponding control groups (704 s, 706 s and 707 s) at 24 h, 7 days and 14 days. 

**Figure 2:**
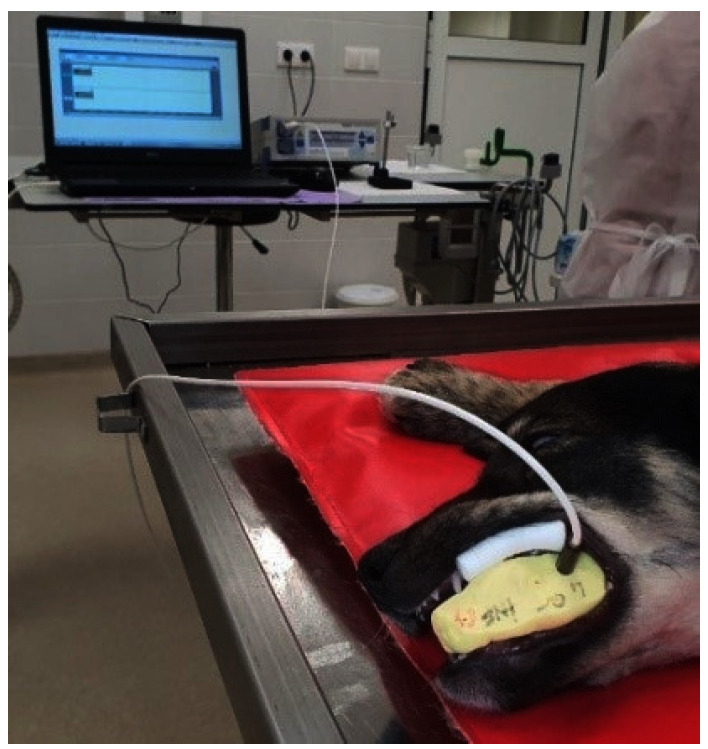
Acquisition of LDF signal using a double silicone impression for the stabilization of the probe and liquid rubber dam for the isolation of the pulpal signal from the periodontal contaminating signal.

**Figure 3:**
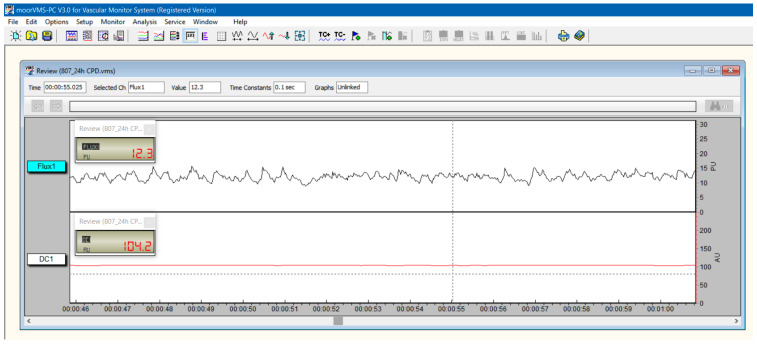
LDF pulp signal recorded in tooth 807, 24 h after DPC (T1).

The LDF recordings of the dental pulp flow were carried out using the Moor Instruments MoorLab VMS-LDF2 monitor (Moor Instruments Ltd., Axminster, UK). The instrument uses laser radiation generated by a semi-conductor laser diode operating at a wavelength of 780 + 10 nm and a maximum accessible power of 1.6 mW. The selected bandwidth for the recorded LD signal was 20 Hz–20 kHz, and the sampling frequency was 40 Hz. Calibration was performed according to the manufacturer’s instructions. The average reading time was 1 min/tooth. The results were recorded and analyzed using MoorSoft MoorLab V2.01 software. A double silicone impression (Optosil Comfort Putty and Activator Universal Plus, Kulzer GmbH., Hanau, Germany) was fixed perpendicularly on the buccal cervical surface of the teeth for stabilization of the probe (Figure 2). Liquid rubber dam (SDI Gingival Barrier, SDI Ltd., Chicago, IL, USA) was used for the supplementary isolation of the gingival tissue. The laser Doppler signal acquisition was performed according to our previous studies [23,24,25]. The physical parameters assessed were flux, expressed in perfusion units (PU), and DC, which indicates the position of the optical probe, reflecting its mechanic stability at the level of the reading area (Figure 3). The recordings were carried out on the treated teeth and their corresponding control groups at T0 and T1.

Radiographical examinations were performed at T0 and T3 in the Department of Radiology of USAMVB. Computer tomography (CT) scans were also carried out at T3, using a Siemens SOMATOM Definition AS 64 scanner (Siemens AG, Erlangen, Germany) and SYNGO Examination integrated software (Siemens AG, Erlangen, Germany) for pulp density measurements. The measurements were performed on axial 0.6 mm-thick slices, at the same level, in the DPC teeth and their corresponding control groups. 

### 2.3. Histologic Examination

The histologic examination was performed according to a modified protocol (Nowicka et al., 2013). The samples were demineralized using Shandon TBD-2 decalcifying solution (Fisher Scientific, Gothenburg, Sweden) and embedded in paraffin. Five-micron-thick serial sections were cut in the bucco-lingual plane of the teeth, stained with hematoxylin-eosin (HE) and then assessed by an experienced oral pathologist (M.M.) using the optical microscope Olympus BX46 (Olympus Co., Tokyo, Japan), with an integrated camera system for image acquisition—at 200×, 100× and 40× magnification. The samples were divided into three histomorphological sample batches (CH, MTA, Biodentine^TM^) and analyzed using the following criteria: pulp inflammation (type, intensity and extension), the amount of hard tissue formation at the interface of the capping material (continuity, morphology and thickness) and the odontoblast cell layer. Each section was scored using a 1–4 numeric scale, with 1 representing the most desired result and 4 representing the least desired result, as follows: for the type of pulp inflammation: 1 = no inflammation, 2 = chronic inflammation, 3 = acute and chronic inflammation and 4 = acute inflammation; for the intensity of pulp inflammation: 1 = absent or very few inflammatory cells, 2 = mild, defined as an average of <10 inflammatory cells, 3 = moderate, defined as an average of 10–25 inflammatory cells and 4= severe, defined as an average >25 inflammatory cells; for the extension of pulp inflammation: 1 = absent, 2 = mild, defined as inflammatory cells only next to dentin bridge or area of pulp exposure, 3 = moderate, defined as inflammatory cells observed in part of coronal pulp (in one-third or more of the coronal pulp or in the mid pulp) and 4 = severe, defined as all coronal pulp is infiltrated or necrotic; for the odontoblastic layer: 1 = palisade pattern of cells, 2 = presence of odontoblast cells and odontoblast-like cells, 3 = presence of only odontoblast-like cells and 4 = absent; for the continuity of the dentinal bridge: 1 = complete dentin bridge formation, 2 = partial/incomplete dentin bridge formation extending to more than one-half of the exposure site but not completely closing the exposure site, 3 = initial dentin bridge formation extending to not more than one-half of the exposure site and 4 = no dentin bridge formation; for the morphology of the dentinal bridge: 1 = dentin or dentin associated with irregular hard tissue, 2 = only irregular hard tissue deposition, 3 = only a thin layer of hard tissue deposition and 4 = no hard tissue deposition [26]. 

### 2.4. Statistical Analysis

The data obtained from the histopathological evaluation and the pulp vitality recordings were processed with the statistical software R Version 4.1.2 2021, RStudio 2022.02.3 + 492 “Prairie Trillium” and JAMOVI Version 1.8.3.0. For statistical analyses, both parametric and non-parametric tests were applied, according to the results of the Shapiro–Wilk test of normality. Parametric tests including Independent Samples *t*-Test and ANOVA and non-parametric tests including Kruskal–Wallis and Mann–Whitney I were used for the comparative evaluation of pulp-testing results before and after DPC and for comparative histological analysis. A *p* value < 0.05 was considered statistically significant. In some situations, we also considered the effect size as a qualitative assessment of the effects (values > 0.5 can be considered as a medium to large effect).

## 3. Results

### 3.1. Preoperative Research

The preoperative radiographical images revealed the normal development of the primary teeth in relation to the adjacent structures (Figure 3). The following developmental stages were noted in the selected primary teeth: second and third premolars within the stability stage (II/III), with no evidence of root resorption, and canines within the first phase of resorption stage (III/III) with less than one-third of the root resorbed. 

PO recordings were carried out at T0 on all selected healthy primary teeth (upper and lower canines and premolars) to serve as reference data. The mean values recorded for pulpal SpO₂ were as follows: 90.9% ± 2.87 SD for canines (C), 89.7% ± 3.21 SD for second premolars (P2) and 89.1% ± 3.13 SD for third premolars (P3)—Table 1. 

The data were statistically processed using the ANOVA test, which revealed no significant differences (*p* = 0.054, >0.05) between the three tooth categories (C, P2 and P3). However, a post hoc analysis of the data using the Tukey HSD test shows a larger difference between C and P3, (*p_tuke_*_y_ = 0.048) with a Cohen’s effect size of 0.600 (if >0.5, the effect can be considered medium to significant). Independent Samples *t*-Test was applied to compare values of upper vs. lower teeth, and no significant differences were detected (*p* = 0.747, >0.05). 

LDF recordings were carried out at T0 only on lower healthy primary canines and premolars (reduced number of teeth due to lengthy procedure). The data were statistically processed using the ANOVA test, which revealed significant differences (*p* = 0.029, < 0.05) between the three tooth categories (C, P2 and P3). The Tukey HSD post hoc comparison showed the most significant differences (*p_tukey_* = 0.022) between P2 and P3.

### 3.2. Post-Operative Research

#### 3.2.1. Imagistic Research

**Figure 4:**
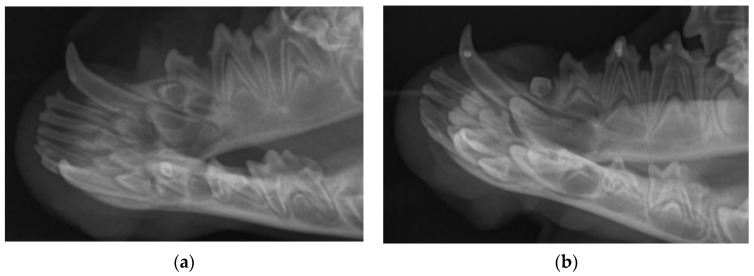
Radiographical image of the right mandible: (**a**) prior to DPC, (**b**) 14 days after DPC of teeth 804, 806 and 807.

The radiographical images showed no periapical lesion in any of the treated teeth (Figure 4) at 14 days after the DPC. However, a slight progression in the physiologic root resorption of the primary canines was noted.

**Figure 5:**
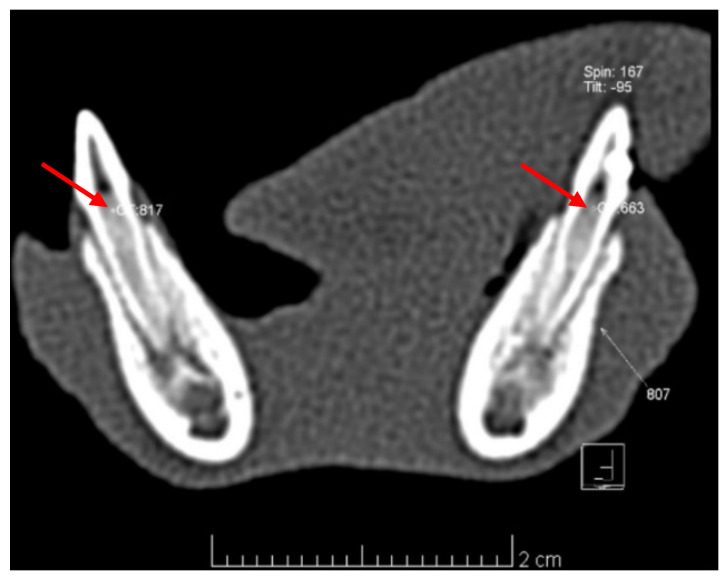
Axial CT slice of 0.6 mm thickness for teeth 807 (14 days after Biodentine^TM^ DPC) and 707 (control) with pulp density measurements (expressed in HU), *red arrow:* conducted at the same level for both teeth (CT: 663 HU in tooth 807 and CT:817 HU in tooth 707).

Pulp density measurements performed comparatively on the axial CT images in the DPC teeth and their corresponding control groups (Figure 5) revealed the following average values (Hounsfield units, HU): 804 = 328.87 HU, 704 = 506.25 HU; 806 = 579.12 HU, 706 = 698.37 HU; 807 = 694.37 HU, 707 = 622.62 HU (Table 2). According to the reference intervals for normal/inflamed/necrotized pulp by Pramanik et al. (2016) [27] and Marzook et al. (2010) [28], six out of eight teeth in the CH group (804) and two out of eight teeth in the MTA group (806) exhibit pulp inflammation, while the Biodentine^TM^ group (807) and all control groups (704, 706 and 707) exhibit normal pulp.

#### 3.2.2. Vitality Tests

**Figure 6:**
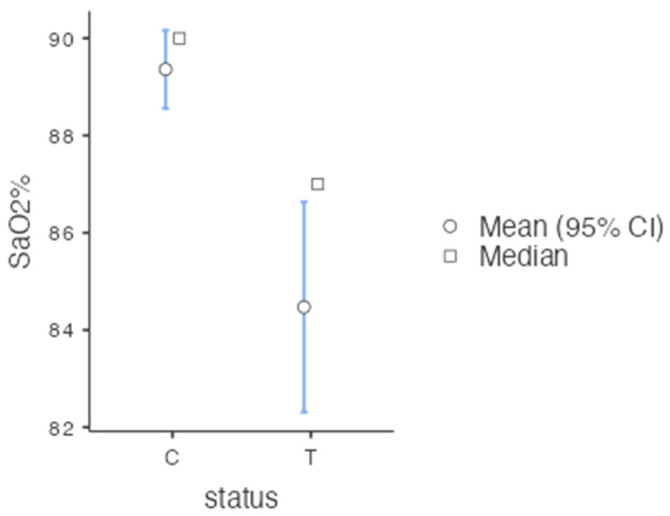
SpO_2_% in the treatment vs. control groups (T1–T3).

Statistical analysis of post-operative SpO₂ recordings revealed that there are significant differences (*p* = 0.002, <0.05) between the treatment vs. control groups (T1–T3), with an effect size of 0.295 (Mann–Whitney non-parametric test)—Figure 6; there are no significant differences (*p* = 0.257, >0.05) between the three treatment groups overall (T1–T3) and also no significant differences between the groups; no significant differences were observed at T1 (*p* = 0.559, >0.05) nor at T3 (*p* = 0.810, >0.05) (One-Way ANOVA and Kruskal–Wallis non-parametric tests).

Statistical analysis of LDF recordings revealed that there are significant differences between materials (*p* = 0.001, <0.05) and also significant differences (*p* = 0.002, <0.05) between the treatment and control groups at T1 (Independent Samples *t*-Test).

#### 3.2.3. Histologic Analysis

**Figure 7:**
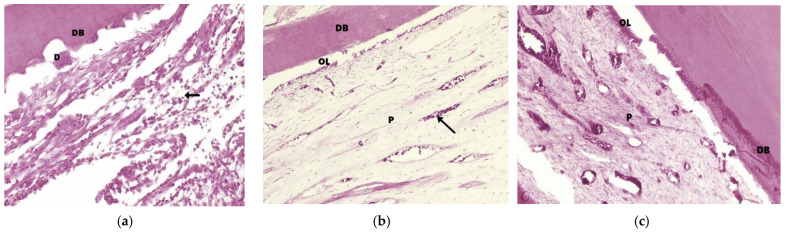
Dental pulp of dog primary teeth capped with different biomaterials (HE coloration): (**a**) CH– arrow = marked acute and chronic severe pulpal inflammation; D = dentin flakes; DB = dentinal bridge (100× magnification). (**b**) MTA—*p* = normal pulp tissue; arrow = minimal inflammatory reaction; hyperemic blood vessels; DB = dentinal bridge; OL = odontoblastic layer (100× magnification). (**c**) Biodentine^TM^—*p* = normal pulp tissue; DB = dentinal bridge; OL = odontoblastic layer (200× magnification).

The histologic evaluation of teeth confirmed the presence of vital pulp tissue in all specimens, exhibiting different degrees of inflammation. MTA and Biodentine^TM^ were well tolerated by the pulp tissue, while in CH-capped teeth, a persistent inflammation was noted (Figure 7). There was evidence of moderate and severe acute and chronic pulpal inflammation in eight out of eight specimens in the CH group, mild and moderate acute inflammation in six out of eight specimens in the MTA group and mild acute inflammation in two specimens out of eight specimens from the Biodentine^TM^ group. Odontoblast and odontoblast-like cells were discovered adjacent to the dentinal bridge in most specimens from the MTA and Biodentine^TM^ groups, and in case of the CH group, only two specimens presented odontoblast-like cells. Complete dentinal bridge formation was not observed in any of the specimens from the three groups.

Results of the histologic evaluation of all specimens from the three groups, according to the numeric 1–4 scale, are summarized in Table 3.

Statistical analysis of the histologic evaluation criteria revealed significant differences between the responses of teeth to the three biomaterials (*p* < 0.001, <0.05, ANOVA statistical test). Detailed Tukey HSD post hoc comparisons between materials revealed the highest statistically significant differences between the Biodentine^TM^ and CH groups (*p_tukey_* < 0.001), significant differences between the MTA and CH groups (*p_tukey_* = 0.001) and no significant differences between the Biodentine^TM^ and MTA groups *(p_tukey_* = 0.712).

## 4. Discussion

This study assesses the pulpal status in primary teeth, aiming to determine whether a small pulse-oximetry nasal alar sensor can be used in a dental setting (and possibly serve as a basis for the development of a customized dental sensor) for monitorization of pulp conditions in both healthy and pulp-capped teeth. LDF, CT, radiographical and histological examinations were used to assess and compare the results. The findings of our study indicate that pulse-oximetry can be a valuable aid in monitoring pulp statuses in primary teeth, having correctly identified vitality in all healthy and treated teeth (accordance between PO, LDF, CT densitometry and histologic data); the first null hypothesis stating that pulse oximetry cannot provide a confirmation of the pulp status (vital/non-vital) in primary teeth following direct pulp capping was, therefore, rejected. The study presents comprehensive histologic data about the pulpal response induced by three DPC materials—CH, MTA and Biodentine^TM^ (regularly used by pediatric dentists in practice). Significant statistical differences between the responses of teeth to the three biomaterials were registered; consequently, the second null hypothesis stating that that there is no statistically significant difference between the pulpal response generated by calcium hydroxide and calcium silicate-based materials was also rejected. While the outcome for the two calcium-silicate cements (MTA and Biodentine^TM^) was similar—minimal pulpal inflammation, with no statistically significant difference between the two materials—the outcome for CH was poorly marked acute and chronic inflammation, with statistically significant differences between Biodentine^TM^ and CH and also between the MTA and CH groups. 

The study was carried out on primary teeth from dogs, which have a life span of approximately five months, with an eruption at about three weeks and shedding at around six months of age [29]. Dogs have been used in numerous studies as animal models for studying the physiopathology of dental pulp. Canines and the premolars of dogs are anatomically close to those of humans. Similarly, dogs share many biochemical and physiologic characteristics with humans, particularly concerning the immune system [30]. As far as we know, only one study in the literature (Riehl et al. 2016) used PO to detect the blood flow in vital canine teeth [15]. However, this study was carried out in intact permanent canines without investigating the pulpal status after pulp-capping procedures. 

The majority of existing PO studies was carried out on anterior teeth [14], due to limitations imposed by the size of the sensor. In our study, we selected, for the pulse-oximetry vitality testing, the nasal alar SpO₂ sensor because the small size of the sensor makes it appropriate for dental use, even in the posterior area of the oral cavity, ensuring a satisfactory tooth-fit. The sensor produces a strong, consistent signal, even in patients with poor perfusion [31]. The data we obtained from PO testing were confronted with data from a second vitality test—LDF (with a standardized technique), CT densitometry of the pulp tissue and histologic analysis—which, in case of the dental pulp, is the reference standard for measuring the diagnostic accuracy of a pulp test [9]. All the afore-mentioned methods confirmed vitality in all teeth at 14 days post-DPC. PO testing after DPC registered values of SpO_2_ ranging between 82.2% and 86.8% in the treated teeth. According to the literature, average SpO_2_ values of healthy teeth are 84.94%–89.29% for central and lateral incisors, 89.20% for canines [14], 86.20% for premolars [32], and 85.09% for molars [33]. Minimum oxygen saturation levels in healthy pulp were identified at around 77.52% [14]. Anusha et al. reported oxygen saturation levels for pulpal diseases: 85.4%—reversible pulpitis 81.6%—irreversible pulpitis; 70.7%—pulp necrosis (94.6%—positive control, healthy teeth; 0%—negative control, endodontically treated teeth) [34]. All these data were recorded, however, in permanent teeth. In primary teeth, the values of SpO_2_ are influenced in some degree by the physiologic root resorption. There are conflicting data in the literature on this matter. Komatsu et al. (2007) found that the pulpal blood flow of primary teeth showed a tendency to decrease with age, due to the morphological changes in the blood vessels in the pulp [35], while Karayilmaz et al. (2011) reported an increase in pulpal blood flow, which is attributed to the progressive apical enlargement caused by physiological root resorption [36]. Given the heterogeneity of the available data on dental SpO_2_, a conclusion regarding the presence of pulp inflammation in the treatment groups from our study cannot be drawn. It can be speculated that another factor with possible implications for PO readings could be the presence of pulp capping and filling materials, which exhibit different light-scattering patterns in comparison to the hard dental tissues. 

Our statistical analysis of the PO recordings revealed that there were no significant differences between the three capping materials, while with LDF testing, there was a statistically significant difference between materials, confirmed by CT densitometry and histological analysis. As with PO, controversies exist in the literature regarding the use of LDF for pulpal blood flow detection as well. On the other hand, some studies found LDF to be an accurate vitality testing method to assess the pulpal vascularity changes of human primary teeth [35], while others reported the opposite [37]. Moreover, the LDF procedure is a lengthy and more sensitive technique compared to conventional pulp vitality tests [9,38], which makes it improper for routine use in children; however, it is reserved for research purposes and particular clinical situations such as trauma to the anterior teeth [39] or “dens invaginatus” [40]. In our study, LDF testing was limited to T0 and T1 (tests at T2 and T3 are not completed) due to the rapid growth of the canine specimens in the two-week time span of the study, which negatively impacted the adaptation of the stabilizing silicone impression. It was decided, therefore, not to manufacture a new impression in order to refrain from altering the reading parameters by placing the probe in a different position. Using the current technique presented in this article, the LDF is not suitable for vitality monitorization of canine primary teeth for time periods longer than 24 h. 

The CT scans performed at T3 provided data about the density of the pulp tissue in the treated teeth and their corresponding control groups. CT allows the precise three-dimensional evaluation of anatomic structures and a direct measurement of tissue density expressed in Hounsfield units (HU). HU is the standard numeric value representing the relative density of body tissues according to a calibrated grey-level scale based on values for air (−1000 HU), water (0 HU) and bone density (+1000 HU) [41]. The density of the dental pulp ranges from −461.5 HU to −170 HU in the case of pulp necrosis, 243.5 HU −396 HU in pulp inflammation and 465 HU −775 HU when the pulp is healthy (with an average of 520 HU for coronal pulp and 892 HU for radicular pulp) [27,28]. The values obtained in our study are indicative for pulp inflammation in most of the CH-capped teeth, and all Biodentine^TM^-capped teeth and all the teeth in the three corresponding control groups are indicative for healthy pulp in most of the MTA-capped teeth. The data are consistent with histologic findings. Although accurate, the routine use of CT for diagnostic purpose of the pulp status is not recommended in children due to radiation concerns [42]. 

Histological examination is regarded in the literature as the gold standard (the best available method against which the performance of other diagnostic tests is evaluated) for dental pulp [9,43]. The histological analysis in our study was aimed primarily at confirming the pulp status of the teeth from the three treatment groups. The extraction of the treated dental units was performed at two weeks post-DPC to avoid the overlapping of physiological root resorption. The results confirmed the presence of vital pulp in all specimens and revealed further data about the inflammatory status present in some of the specimens. Overall, the least amount of evidence for inflammation was found in the Biodentine^TM^ group, while in the CH group, markers of acute and chronic inflammation were present in all specimens, with moderate and severe extension and intensity. Dentinal bridge formation was initiated in specimens from all three groups. Nevertheless, this finding must be interpreted with care, as it can be a sign of healing or a reaction to irritation [26]. In this case, given that the teeth were extracted only two weeks after pulp capping, it is premature to consider it a sign of healing, especially in case of CH specimens, where pulp inflammation is present. The results of the histological analysis from this study are in accordance with previous studies [44,45]. 

The limitations of the present study are the low number of primary teeth included in the experiment, the rapid growth of young canine subjects (aged three months at the debut of the study) and the lack of dedicated devices/sensors for dental pulp vitality testing (which may have affected the measurements, due to the imperfect adaptation of the sensor to the teeth, instability of the hand-held sensor and the different optical properties of the tissues and filling/capping materials).

## 5. Conclusions

Within its limitations, the present study confirms, with histologic means, the diagnostic value of the PO evaluation of pulpal statuses in primary teeth after pulp-capping procedures in a canine model. However, various degrees of pulpal inflammation elicited by different pulp-capping materials seem not to correlate with the obtained PO values. LDF, in its current version, is not a suitable pulp testing method for the monitorization of vitality in primary canine teeth for a longer period due to technique-related limitations. The results of our study could contribute to the improvement of pulp-testing methods and their applications in pediatric dental practice. Future research should be focused on developing a dedicated PO sensor for dental use and establishing SpO_2_ reference parameters for all dental categories, as well as for treated teeth. The influence of different filling materials (with optical properties that differ from those of the hard dental structures) on the SpO_2_ readings should be acknowledged in detail.

## Figures and Tables

**Table 1:** Mean SpO_2_ values recorded in healthy primary canines and premolars.

**SpO_2_**	**Tooth Position**	**Tooth Type**	**Mean**	**Median**	**SD**
	Lower	C	91.8	91.0	2.62
		P2	89.6	90.5	3.34
		P3	88.6	89.5	3.33
	Upper	C	90.1	90.5	2.93
		P2	89.7	90.0	3.18
		P3	89.6	90.0	2.94

**Table 2:** Average values of pulp density (HU) measured on CT scans in all treated and control groups.

Treated/Control Group	Average Values of Pulp Density
804/704	328.87 HU/506.25 HU
806/706	579.12 HU/698.37 HU
807/707	694.37 HU/622.62 HU

**Table 3:** Modal values of the different criteria characterizing the histologic features of the three DPC biomaterials, according to the scores (1–4).

Biomaterial	Criteria			All Criteria
	Inflammation	Odontoblastic Layer	Dentinal Bridges	
Ca(OH)_2_	3	4	2	4
MTA	2	1	3	2
Biodentine^TM^	1	1	3	1

## Data Availability

Not applicable.

## References

[1] American Academy of Pediatric Dentistry (2020). The Reference Manual of Pediatric Dentistry.

[2] Cushley S., Duncan H.F., Lundy F.T., Nagendrababu V., Clarke M., Karim I.E. (2022). Outcomes reporting in systematic reviews on vital pulp treatment: A scoping review for the development of a core outcome set. Int. Endod. J..

[3] Chen E., Abbott P.V. (2009). Dental Pulp Testing: A Review. Int. J. Dent..

[4] Gopikrishna V., Pradeep G., Venkateshbabu N. (2009). Assessment of pulp vitality: A review. Int. J. Paediatr. Dent..

[5] Jafarzadeh H., Abbott P.V. (2010). Review of pulp sensibility tests. Part I: General information and thermal tests. Int. Endod. J..

[6] Vaghela D., Sinha A. (2011). Pulse oximetry and laser doppler flowmetry for diagnosis of pulpal vitality. J. Interdiscip. Dent..

[7] Sharma A., Madan M., Shahi P., Sood P., Shahi N. (2015). Comparative Study of Pulp Vitality in Primary and Young Permanent Molars in Human Children with Pulse Oximeter and Electric Pulp Tester. Int. J. Clin. Pediatr. Dent..

[8] Mainkar A., Kim S.G. (2018). Diagnostic Accuracy of 5 Dental Pulp Tests: A Systematic Review and Meta-analysis. J. Endod..

[9] Mejàre I.A., Axelsson S., Davidson T., Frisk F., Hakeberg M., Kvist T., Norlund A., Petersson A., Portenier I., Sandberg H. (2012). Diagnosis of the condition of the dental pulp: A systematic review. Int. Endod. J..

[10] Grabliauskienė Ž., Zamaliauskienė R., Lodienė G. (2021). Pulp vitality testing with a developed universal pulse oximeter probe holder. Medicina.

[11] Janani K., Ajitha P., Sandhya R., Subbaiyan H., Jose J. (2020). Efficiency of new custom-made pulse oximeter sensor holder in assessment of actual pulp status. J. Fam. Med. Prim. Care.

[12] Sharma D.S., Mishra S., Banda N.R., Vaswani S. (2019). In vivo evaluation of customized pulse oximeter and sensitivity pulp tests for assessment of pulp vitality. J. Clin. Pediatr. Dent..

[13] Almudever-Garcia A., Forner L., Sanz J.L., Llena C., Rodríguez-Lozano F.J., Guerrero-Gironés J., Melo M. (2021). Pulse oximetry as a diagnostic tool to determine pulp vitality: A systematic review. Appl. Sci..

[14] Lambert P., Miguens S.A.Q., Solda C., Sganzerla J.T., Reichert L.A., Estrela C., Barletta F.B. (2020). Reference values for pulp oxygen saturation as a diagnostic tool in endodontics: A systematic review and meta-analysis. Restor. Dent. Endod..

[15] Riehl J., Hetzel S.J., Snyder C.J., Soukup J.W. (2016). Detection of pulpal blood flow in vivo with pulse oximetry in dogs. Front. Vet. Sci..

[16] Duncan H.F. (2022). Present status and future directions—Vital pulp treatment and pulp preservation strategies. Int. Endod. J..

[17] Hanna S.N., Perez Alfayate R., Prichard J. (2020). Vital Pulp Therapy an Insight Over the Available Literature and Future Expectations. EUR Endod. J..

[18] Li Z., Cao L., Fan M., Xu Q. (2015). Direct Pulp Capping with Calcium Hydroxide or Mineral Trioxide Aggregate: A Meta-analysis. J. Endod..

[19] Mohammadi Z., Dummer P.M.H. (2011). Properties and applications of calcium hydroxide in endodontics and dental traumatology. Int. Endod. J..

[20] Emara R., Elhennawy K., Schwendicke F. (2018). Effects of calcium silicate cements on dental pulp cells: A systematic review. J. Dent..

[21] Stringhini Junior E., dos Santos M.G.C., Oliveira L.B., Mercadé M. (2019). MTA and Biodentine for Primary Teeth Pulpotomy: A Systematic Review and Meta-Analysis of Clinical Trials. Clin. Oral Investig..

[22] Coll J.A., Seale N.S., Vargas K., Marghalani A.A., Al Shamali S., Graham L. (2017). Primary Tooth Vital Pulp Therapy: A Systematic Review and Meta-analysis. Pediatr. Dent..

[23] Miron M.-I., Dodenciu D., Sarbescu P., Canjau S., Ardelean L., Rusu L.C., Todea C. (2012). Condensation Silicones and Light-curing Resin Used within a Laser Doppler Pulp Vitality Testing Method. Mater. Plast..

[24] Miron M., Lungeanu D., Ciora E., Ogodescu E., Todea C. (2020). Using laser-doppler flowmetry to evaluate the therapeutic response in dentin hypersensitivity. Int. J. Environ. Res. Public Health.

[25] Miron M.I., Barcutean M., Luca R.E., Todea C.D., Tudor A., Ogodescu E. (2022). The Effect of Changing the Toothbrush on the Marginal Gingiva Microcirculation in the Adolescent Population—A Laser Doppler Flowmetry Assessment. Diagnostics.

[26] Nowicka A., Lipski M., Parafiniuk M., Sporniak-Tutak K., Lichota D., Kosierkiewicz A., Kaczmarek W., Buczkowska-Radlińska J. (2013). Response of human dental pulp capped with biodentine and mineral trioxide aggregate. J. Endod..

[27] Pramanik F., Firman R.N., Oscandar F., Epsilawati L. (2016). Normal, inflammation and necrosis pulp radiograph image using 3D cone beam computed tomography. Padjadjaran J. Dent..

[28] Marzook H.A., Elhawary Y.M., El-gindy A.A., Radwan L.R.S. (2010). Cone Beam Computed Tomography Voxel Values of Dental Structures. Egypt. Dent. J..

[29] Igna C., Sabău M., Șereș M., Dascălu R., Igna C. (2008). Stomatologie Veterinară.

[30] Aubeux D., Renard E., Pérez F., Tessier S., Geoffroy V., Gaudin A. (2021). Review of Animal Models to Study Pulp Inflammation. Front. Dent. Med..

[31] Schallom M., Prentice D., Sona C., Arroyo C., Mazuski J. (2018). Comparison of nasal and forehead oximetry accuracy and pressure injury in critically ill patients. Heart Lung.

[32] Estrela C.R.A.C., Serpa G.C., Alencar A.H.G., Bruno K.F., Barletta F.B., Felippe W.T., Estrela C.R.A.C., Souza J.B. (2017). Oxygen saturation in the dental pulp of maxillary premolars in different age groups—Part 1. Braz. Dent. J..

[33] Estrela C., Oliveira K.S.A., Alencar A.H.G., Barletta F.B., Estrela C.R.A., Felippe W.T. (2017). Oxygen saturation in the dental pulp of maxillaryandmandibularmolars-part 2. Braz. Dent. J..

[34] Anusha B., Madhusudhana K., Chinni S.K., Paramesh Y. (2017). Assessment of pulp oxygen saturation levels by pulse oximetry for pulpal diseases -a diagnostic study. J. Clin. Diagn. Res..

[35] Komatsu H., Ikawa M., Mayanagi H. (2007). Age-related changes of pulpal blood flow in primary teeth measured by laser Doppler blood flowmetry. Pediatr. Dent. J..

[36] Karayilmaz H., Kirzioǧlu Z. (2011). Evaluation of pulpal blood flow changes in primary molars with physiological root resorption by laser doppler flowmetry and pulse oximetry. J. Clin. Pediatr. Dent..

[37] Ghouth N., Duggal M.S., Kang J., Nazzal H. (2019). A Diagnostic Accuracy Study of Laser Doppler Flowmetry for the Assessment of Pulpal Status in Children’s Permanent Incisor Teeth. J. Endod..

[38] Roeykens H., De Moor R. (2011). The use of laser Doppler flowmetry in paediatric dentistry. Eur. Arch. Paediatr. Dent..

[39] Belcheva A., Shindova M., Hanna R. (2021). Efficacy of Laser Doppler Flowmetry, as a Diagnostic Tool in Assessing Pulp Vitality of Traumatised Teeth: A Split Mouth Clinical Study. J. Pers. Med..

[40] Lee H.N., Yan D.Y., Huang C.Y., Chen S.C., Pan C.Y., Jeng J.H., Chen Y.K., Chuang F.H. (2021). Laser Doppler for Accurate Diagnosis of Oehler’s Type III Dens Invaginatus: A Case Report. Appl. Sci..

[41] Silva I.M.D.C.C., De Freitas D.Q., Ambrosano G.M.B., Bóscolo F.N., Almeida S.M. (2012). Bone density: Comparative evaluation of hounsfield units in multislice and cone-beam computed tomography. Braz. Oral Res..

[42] Kühnisch J., Anttonen V., Duggal M.S., Loizides Spyridonos M., Rajasekharan S., Sobczak M., Stratigaki E., Van Acker J.W.G., Aps J.K.M., Horner K. (2019). European Archives of Paediatric Dentistry Best clinical practice guidance for prescribing dental radiographs in children and adolescents: An EAPD policy document. Eur. Arch. Paediatr. Dent..

[43] Alghaithy R.A., Qualtrough A.J.E. (2017). Pulp sensibility and vitality tests for diagnosing pulpal health in permanent teeth: A critical review. Int. Endod. J..

[44] Andrei M., Vacaru R.P., Coricovac A., Ilinca R., Didilescu A.C., Demetrescu I. (2021). The effect of calcium-silicate cements on reparative dentinogenesis following direct pulp capping on animal models. Molecules.

[45] Hamdy M., Fayyad D., Eldaharawy M., Hegazy E. (2018). Physical properties of different Pulp Capping Materials and Histological Analysis of their effect on Dogs’ Dental Pulp Tissue Healing. Egypt. Dent. J..

